# Examining caregiver-adolescent symptom agreement and its influence on treatment outcomes: a quantitative analysis

**DOI:** 10.3389/fpsyg.2025.1676629

**Published:** 2025-12-10

**Authors:** Elizabeth Kroll, Sara Hessdorf, Alyson Brandis, Klint Kanopka, Jonathan Kohlmeier, Hannah Heard, Izabella Zant, Stephanie Stolzenbach, Caroline Fenkel

**Affiliations:** 1 Charlie Health, Inc, Bozeman, MT, United States; 2 Department of Practicum Education and Community Partnerships, New York University, New York, NY, United States; 3 Department of Applied Statistics, Social Science, and Humanities, Steinhardt School of Culture, Education, and Human Development, New York University, New York, NY, United States

**Keywords:** IOP, mental health, depression, anxiety, caregivers, adolescents

## Abstract

**Introduction:**

Caregivers play a critical role in identifying and interpreting adolescent mental health symptoms, yet their perceptions often differ from those of adolescents themselves. Such discrepancies can influence treatment engagement and outcomes, making it essential to understand the factors that affect caregiver–adolescent agreement in symptom reporting.

**Methods:**

This study analyzed baseline depression and anxiety symptom reports from 763 caregiver–adolescent dyads enrolled in a virtual intensive outpatient program. Agreement between adolescent self-reports and caregiver proxy reports was assessed using concordance analyses. Potential moderators included adolescent age, gender identity, race, neurodivergence, caregiver well-being, and family functioning. Additional analyses examined whether agreement predicted treatment outcomes, including symptom improvement and treatment completion.

**Results:**

Overall, caregiver–adolescent agreement was low to moderate (CCC = 0.215). Adolescents identifying as non-binary showed the largest discrepancies, typically rating their symptoms as more severe than their caregivers. Higher family functioning was associated with reduced directional disagreement for depression, though this pattern did not hold for anxiety. Dyads in which caregivers reported higher symptom severity than adolescents demonstrated greater symptom improvement by discharge, while agreement levels did not significantly predict treatment completion.

**Discussion:**

Informant discrepancies were common and clinically meaningful, particularly among gender-diverse adolescents and families reporting lower functioning. Although overall agreement did not predict treatment completion, caregiver perceptions of greater symptom severity were linked to greater reductions in adolescent depression and anxiety over treatment. These findings suggest that incorporating caregiver perspectives into assessment and treatment planning may enhance outcomes, especially in high-acuity virtual care settings. Future research should investigate mechanisms through which caregiver engagement influences adolescent symptom change over time.

## 1 Introduction

### 1.1 Adolescent mental health

The mental health crisis among adolescents and young adults is a significant public health concern, with anxiety and depression being among the most prevalent disorders affecting this demographic (Patel et al., 2007; McGorry and Mei, 2018). The World Health Organization (2024) reports that 4.4% of 10–14-year-olds and 5.5% of 15–19-year-olds experience an anxiety disorder, and an estimated 1.4% of adolescents aged 10–14 years, and 3.5% of 15–19-year-olds experience depression. Within the United States, the Centers for Disease Control and Prevention (2025) state that approximately 13% of adolescents experienced a diagnosed mental or behavioral health condition between 2018 and 2019. For young adults, this figure rises to nearly 25%, reflecting the ongoing challenges they face as they navigate the transition to adulthood.

These conditions may disrupt daily functioning, education, family formation, and damage social relationships (Gustavson et al., 2018). They also can result in life-threatening situations such as suicidal ideation (Windarwati et al., 2022). A study by Hughes et al. (2017) underscores the long-term consequences of untreated mental health disorders, linking early exposure to anxiety and depression with increased risks of substance use, chronic health problems, and poor relationship outcomes in adulthood. They found that those who experienced mental health challenges in adolescence were more likely to experience difficulties in maintaining stable employment and relationships as young adults (Hughes et al., 2017). Children with untreated anxiety can also experience impaired coping such as substance use or suicidal ideation (Bosworth, 2019). Recognizing these multifaceted influences highlights the importance of early identification and intervention, as addressing these factors can significantly improve long-term mental and behavioral health outcomes for adolescents and young adults.

### 1.2 The role of caregivers in adolescent mental health treatment

Caregivers play a crucial role in the mental health of adolescents, as children rely on their caregivers for basic needs, emotional support, and socialization (Center on the Developing Child, 2016). Bowen Family Systems theory frames the family as an interconnected system in which distress or dysfunction in one member can reverberate across the entire unit, sometimes across generations (Erdem and Safi, 2018). Research further supports this system’s view: Panter-Brick et al. (2014) found that caregiver mental health scores were predictive of the scores of their adolescents on comparable assessments, suggesting a direct link between parent and child well-being. Furthermore, greater family functioning was correlated with increased parent satisfaction and self-efficacy which is indicative of greater social support from the caregivers (Angley et al., 2015). Together these findings underscore the importance of considering the caregiver context in both assessment and treatment planning for adolescents.

Beyond their role in shaping mental health outcomes, caregivers also serve as gatekeepers of treatment. Caregivers with self-reported mental health issues tend to assess greater need for their adolescents to receive mental health treatment, and are therefore more likely to encourage treatment. In turn, these adolescents experience more successful treatment, including fewer dropouts and improved treatment outcomes (De Los Reyes et al., 2021). By contrast, caregivers who assess adolescents as having fewer mental health issues are less likely to encourage their child to receive mental health treatment, which may be harmful for those who assess themselves differently than their caregivers (Bajeux et al., 2018).

Caregiver misperception is not uncommon: in a study of young children ages 2–6 who met criteria for a psychiatric diagnosis, only 38.8% were perceived by their parents as needing help (McGinnis et al., 2022). While this sample skews younger, it highlights a persistent challenge across developmental stages, caregiver awareness is essential to recognizing and addressing adolescent needs. As a result mental health care can be delayed which has long been shown to have negative effects on individuals with studies showing increased risk of screening for depression and anxiety (Noel et al., 2025), and increased risk of treatment drop out (Costantini, 2025). For neurodivergent individuals specifically, a delay in diagnosis is associated with poorer mental health in general (Huynh et al., 2024; Holden and Kobayashi-Wood, 2025), and increased medical utilization (Vu et al., 2023).

When caregiver and adolescent views align, treatment is more likely to be timely and effective. However, agreement on symptom severity is often inconsistent. A study examining the level of agreement between parents and adolescents and the need for mental healthcare showed that almost half of either group identified a need for mental health care, but only one third had their needs met (Schnyder et al., 2020). Agreement on whether care was needed was moderate with higher disagreement being linked to the parents’ lack of knowledge of the adolescent’s emotional state. This emphasizes the importance of strong communication and connection between caregivers and adolescents, as well as the effect these relationships have on mental health and mutual understanding.

### 1.3 Self-reported vs. caregiver-reported

Comparing mental health assessments from adolescent clients and their caregivers offers critical insight into the high rate of discrepancies in adolescent mental health reporting (Bajeux et al., 2018). Informant discrepancies not only offer insight into a caregiver-child relationship, but can also impact the course of an adolescent’s mental health treatment. Around 76% of caregiver-child dynamics disagree on a target problem to address during treatment (De Los Reyes and Kazdin, 2005). These disagreements may lead to misaligned treatment goals, diminished therapeutic engagement, or conflicting expectations between the therapist, adolescent, and caregiver.

Informant discrepancies show up for a variety of reasons. Adolescent clients may demonstrate limited objectivity when reporting on themselves, their mental health symptoms, and behaviors, in part due to ongoing identity development and evolving self-awareness (Dang et al., 2020). Additionally, adolescent clients may be more likely to under-report symptoms out of shame or a distrust of authority and how these reports will be used (Tarren-Sweeney, 2019). On the other side, caregivers present their own set of biases when it comes to reporting on adolescent mental health. Caregivers tend to over-report external or behavioral problems, and under-report internal problems for adolescents (Bajeux et al., 2018). As a result, adolescents struggling with internalizing symptoms may not receive appropriate interventions, while externalizing behaviors may be more likely to drive treatment decisions.

Neurodivergent conditions such as ASD and ADHD may influence the level of agreement between caregiver- and adolescent-report, in part because differential symptom observability (e.g., externalizing vs. internalizing behaviors), reduced adolescent self-insight, and altered social-cognitive processes (e.g., perspective taking, awareness of change) can create systematic informant divergences (Lopez-Espejo et al., 2023; McDonald et al., 2016; Ismail et al., 2017).

Gender can also influence these patterns of perception. Moens et al. (2018) found that caregivers are less likely to report behavioral problems in female adolescents compared to males, potentially reflecting gendered expectations about emotional expression and social behavior. These biases are often influenced by the caregivers’ own mental health, parenting styles, and attitudes. Specifically, caregivers with their own mental health symptoms tend to overestimate adolescents’ behavioral problems (Ehrlich et al., 2011). Parenting style also plays a role: punitive or authoritarian parents tend to over-pathologize adolescent behavior, whereas more permissive caregivers may underestimate concerns (Bajeux et al., 2018). In short, discrepancies between adolescent and caregiver mental health reports are not random; they are formed by social and psychological factors that can significantly impact assessment accuracy, treatment planning, and the therapeutic alliance.

### 1.4 Study goal

This study examines the relationship between self-reported and caregiver-reported symptom severity for both anxiety and depression, with the goal of identifying patterns in discrepancies between adolescent and caregiver ratings. Additionally, it explores how these differences may influence treatment outcomes. Based on prior literature, we expected to find that high agreement will significantly predict better outcomes for both anxiety and depression symptoms. Additionally, we expected these findings to be moderated by gender. When interpreting results, readers should consider the unique context of a virtual IOP. Our goal was not to evaluate differences between virtual and in-person settings, but rather to examine caregiver–adolescent agreement in the context of virtual care, where the dynamics of reporting and involvement may differ.

## 2 Methods

### 2.1 Setting

Data was collected from adolescent clients, ages 11–17, admitted to Charlie Health and their caregivers. Charlie Health is a virtual intensive behavioral health outpatient treatment program that treats individuals between the ages of 11 to 50 who present with high acuity mental health conditions. Treatment lasts approximately 9–12 weeks. Each week, programming at Charlie Health consists of 9 h of group sessions, 1 h of individual therapy, and an optional hour of family therapy depending on the client’s needs. While not required, family participation is encouraged.

### 2.2 Ethics statement

This study was reviewed and approved by NorthStar Institutional Review Board (Protocol #NB500297) as secondary research under the Common Rule 45 CFR 46.104(d)(4)(ii).

### 2.3 Data collection

Client self-reported data were collected between October 2023 and January 2025 at intake and discharge from Charlie Health’s treatment program. For intake data, clients were sent an email 3 h prior to their initial admissions assessment, which asked them to fill out the intake survey. For those who did not submit prior to their intake assessment, they were given an intake survey in the first hour of their orientation to group sessions. A Charlie Health staff member joined the group and distributed personalized links to each client. For the small subset of clients who did not complete an intake survey by the end of orientation, emails follow ups were sent with the link for up to 1 week post first appointment. At all points of contact, clients were instructed that the survey was optional and would not impact their admission status.

Discharge surveys were distributed on the clients’ last day in group sessions. Clients were informed that the survey was optional and would not affect their discharge status. If a client missed their final group session, they were emailed and texted a personalized link to the discharge survey and prompted to fill it out with a $5 gift card incentive.

Caregiver data was collected between October 2023 and January 2025 during their client’s first full week of admission. The survey was sent via a routine week 1 email to all primary caregivers listed on clients’ account. Surveys remained open for 1 week. Caregivers filled this survey out on their own time.

Both Client and Caregiver surveys were filled out on a personal computer and were asked to be taken in a confidential space.

In order to be included in the study both an intake survey and caregiver survey had to be submitted. Additionally, to be included in discharge analyses, a discharge survey had to be submitted by the client. There were no additional exclusion criteria.

### 2.4 Measures

#### 2.4.1 Client measures

##### 2.4.1.1 Depression

Client self-reported depression severity was measured with The Patient Health Questionnaire (PHQ-9) (Johnson et al., 2002). This nine-item measure uses a 0–3 scale (not at all, several days, more than half the days, nearly every day) for all questions and produces a final sum score between 0–27 with scores of 5, 10, 15, and 20 representing mild, moderate, moderately severe, and severe depression, respectively. Respondents are asked to recall how often symptoms have impacted them in the 2 weeks prior to the assessment.

##### 2.4.1.2 Anxiety

Client self-reported anxiety severity was measured with The Generalized Anxiety Disorder-7 (GAD-7) (Spitzer et al., 2006). This seven-item measure used a 0–3 (not at all, several days, more than half the days, nearly every day) scale for all questions and produced a sum score between 0 and 21 with score cut offs of 5, 10, and 15 representing mild, moderate, and severe anxiety, respectively. Respondents are asked to recall how often symptoms have impacted them in the 2 weeks prior to the assessment.

##### 2.4.1.3 Discharge reason

Reason for discharge was gathered from administrative data and condensed into routine discharge and non-routine discharge. Routine discharge was decided by a client’s primary therapist and defined as the patient completing treatment plan goals, or the patient stabilizing to such an extent that completion of the treatment process at a less restrictive level-of-care was therapeutically appropriate. All other discharge reasons were collapsed into non-routine indicating that they were discharged prior to completing treatment.

##### 2.4.1.4 Demographics

Client demographics data was collected at intake. Clients were asked to disclose their age, gender, race, and neurodivergent identity all identity questions were multi-select to allow clients to select all answers that matched their preferred identities. Additionally, all questions were optional and clients could opt to not disclose any demographic information. Demographic categories are reported in Table 1.

**Table 1:** Client demographics.

Demographic category	Percentage	*n*
Race		
Black or African American	7.86%	60
White	70.77%	540
Other	9.70%	74
Missing	11.66%	89
Gender		
Female	52.42%	400
Male	30.41%	232
Non-binary/other	10.09%	77
Missing	7.08%	54
Neurodivergent identity		
Autism	2.62%	20
ADHD	22.54%	172
AuDHD	3.15%	24
Other	6.42%	49
None	40.37%	308
Missing	24.90%	190

#### 2.4.2 Caregiver measures

##### 2.4.2.1 Caregiver well-being

Caregiver well-being was collected using the WHO-5 well-being Index. This five-item measure is scored on a 0–5 scale and final scores are calculated by summing all answers and multiplying by four to obtain a score on a scale from 0 to 100 with higher scores indicating higher well-being. A score below 50 is often considered at-risk for depression (Topp et al., 2015).

##### 2.4.2.2 Child depression

Caregivers were asked to assess the severity of their loved one’s depression using the PROMIS Parent Proxy Depressive Symptoms short form (Irwin et al., 2012; Broderick et al., 2013). This six-item measure used a 1–5 scale (never, almost never, sometimes, often, almost always) and sum scores were calculated to obtain a final score between 6 and 30. Caregivers were asked to recall how symptoms impacted their loved one in the 7 days prior.

##### 2.4.2.3 Child anxiety

Caregivers were asked to assess the severity of their loved one’s anxiety symptoms using the PROMIS Parent Proxy Anxiety short form (Irwin et al., 2012; Broderick et al., 2013). This eight-item measure used a 1–5 (never, almost never, sometimes, often, almost always) scale and sum scores were calculated to obtain a final score between 8 and 40. Caregivers were asked to recall how symptoms impacted their loved one in the 7 days prior.

##### 2.4.2.4 Family functioning

Family functioning was assessed using the Family Satisfaction Subscale (FSS) of the Family Adaptability and Cohesion Evaluation Scales (FACES), which evaluates overall satisfaction with various aspects of family dynamics. This eight-item measure asks respondents to rate their satisfaction with family-related factors such as emotional closeness, communication quality, time spent together, conflict resolution, and mutual support on a scale of 1 to 5 (never or almost never, once in a while, sometimes, frequently, almost always or always) with a recall time of 1 month prior to assessment.

Items include prompts such as, “The degree of closeness between family members” and, “Your family’s ability to resolve conflicts.” Responses were rated on a likert scale, with higher scores on a scale of 8 to 40 indicating greater satisfaction with family functioning. The FSS has been used in both clinical and research contexts to capture individuals’ subjective evaluations of family support and cohesion (Olson et al., 2013).

##### 2.4.2.5 Demographics

Caregivers were asked to identify their relationship to the client (Table 2).

**Table 2:** Caregiver and client relationships.

Caregiver relationship	Percentage	*n*
Parent	93.71%	715
Grandparent	3.54%	27
Other direct relation	1.18%	9
Legal guardian–non family	0.92%	7
Missing	0.66%	5

### 2.5 Data prep

All data were pulled from their respective locations and merged based on unique client IDs. Client data was deleted from the data set if there was no corresponding caregiver survey. Both client self-reported data and parent proxy scales for depression and anxiety were then standardized using z-score standardization. This process transforms raw scores into standardized values based on the mean and standard deviation of the sample, allowing for direct comparison between measures that originally use different scoring systems.

Level of agreement on depression and anxiety symptoms was calculated by subtracting the client self-reported scaled score from the caregiver proxy scaled score. Negative scores indicated that clients rated their symptoms as more severe than their caregivers; a score of 0 indicated perfect agreement, and positive scores indicated that caregivers scored client symptoms as more severe than the client themselves.

To enable stable analyses, response categories were collapsed into the largest conceptually coherent groupings. This approach reduced small cell sizes while preserving the most meaningful distinctions in the data. Additionally, to enable analyses that required mutually exclusive categories, mutli-select responses were recoded into an “other” category across all demographics.

All analyses were conducted in R (Version 4. X. X; R Core Team, 2013) using the following packages: *dplyr*, *ggplot2*, *ggrepel*, *segmented*, and *rms* (Wickham et al., 2023; Wickham, 2023; Slowikowski, 2023; Muggeo, 2022; Harrell, 2023).

### 2.6 Data analysis strategy

To assess general trends in symptom agreement, concordance correlation coefficients (Lin, 1989) were estimated between client-reported and caregiver-reported depression (PHQ-9 vs. PROMIS Parent Proxy Depression) and anxiety (GAD-7 vs. PROMIS Parent Proxy Anxiety) scores. Bland–Altman analyses were performed to assess variation in agreement across the range of observed symptom severities (Bland and Altman, 1986).

To determine whether client demographics were associated with symptom agreement, a linear regression was conducted to test the relationship between client age and agreement scores for depression and anxiety. Additionally, one-way ANOVAs were performed to assess whether client gender, race, and neurodivergent identity impacted agreement scores, with Bonferroni corrections applied to adjust for multiple comparisons.

Next, the relationship between family functioning and symptom agreement was explored through a series of linear and logistic regressions. Simple linear regressions examined whether family functioning predicted overall symptom agreement for depression and anxiety. Additional models assessed the relationship between family functioning and magnitude of agreement (absolute difference regardless of direction) and family functioning and disagreement direction (whether caregivers rated symptoms higher than clients). Parallel analyses were conducted to investigate whether caregiver well-being was similarly associated with symptom agreement patterns.

To evaluate the impact of symptom agreement on treatment outcomes, linear regressions were conducted to determine whether agreement scores at intake predicted depression and anxiety severity at discharge. Additionally, logistic regressions assessed whether agreement level (overall score, absolute discrepancy, or direction of disagreement) influenced treatment completion (routine vs. non-routine discharge). Finally, based on prior analyses identifying age, gender, and family functioning as significant predictors of agreement, multiple linear regression models were used to determine whether these variables moderated the relationship between agreement scores and post-treatment symptom severity.

## 3 Results

### 3.1 Descriptives

#### 3.1.1 Demographics

Data were collected from 763 client and caregiver duos between October 2023 and January 2025, which represents approximately 5% of the eligible families within this time period (*n* = 16,797) Within this sample, the majority of clients identified as white (70.77%), female (52.42%), and neurotypical (40.37%). Within the caregiver sample, the majority of respondents identified themselves as the client’s parent (93.71%). Additional demographic information can be found in Tables 1, 2.

At intake, clients presented with moderate depression (PHQ-9 M = 13.10, 95% CI [12.60, 13.57]) and anxiety (GAD-7 M = 10.73, 95% CI [10.37, 11.20]). Internal consistency was excellent for both the PHQ-9 (*α* = 0.88) and GAD-7 (α = 0.90) at intake, consistent with the literature. Clients who reported self-harm behavior (38.76%) reported an average of 6.42 days of self-harm in the 30 days leading up to admission. Comparatively, caregiver proxy scores were moderately high for depression (*M* = 19.46, 95% CI [19.10, 19.82]) and moderate for anxiety (*M* = 22.27, 95% CI [21.84, 22.71]). By discharge, clients reported mild depression (*M* = 6.44, 95% CI [5.76, 7.11]) and mild anxiety (*M* = 5.10, 95% CI [4.53, 5.67]), and internal consistency remained high (PHQ-9 α = 0.89; GAD-7 α = 0.91).

On the WHO-5 Well-Being Index, caregivers self-reported an average score of 50.07 (95% CI [48.51, 51.30]), placing them just above the clinical threshold commonly used to flag risk for depression. This score reflects the caregiver’s own well-being, not their perception of the client’s mental health. Approximately 44% (*n* = 398) of caregivers fall below that 50 point threshold indicating poor well-being in almost half of the sample. Internal consistency remained high for the items in the WHO-5 (α = 0.88), consistent with the literature on the WHO-5. Finally, caregivers reported moderately high family functioning (*M* = 29.03, 95% CI [28.62, 29.43]) and the FACES measures showed high internal consistency within this sample (α = 0.87).

### 3.2 Caregiver-child agreement

#### 3.2.1 Overall agreement rates

**Figure 1:**
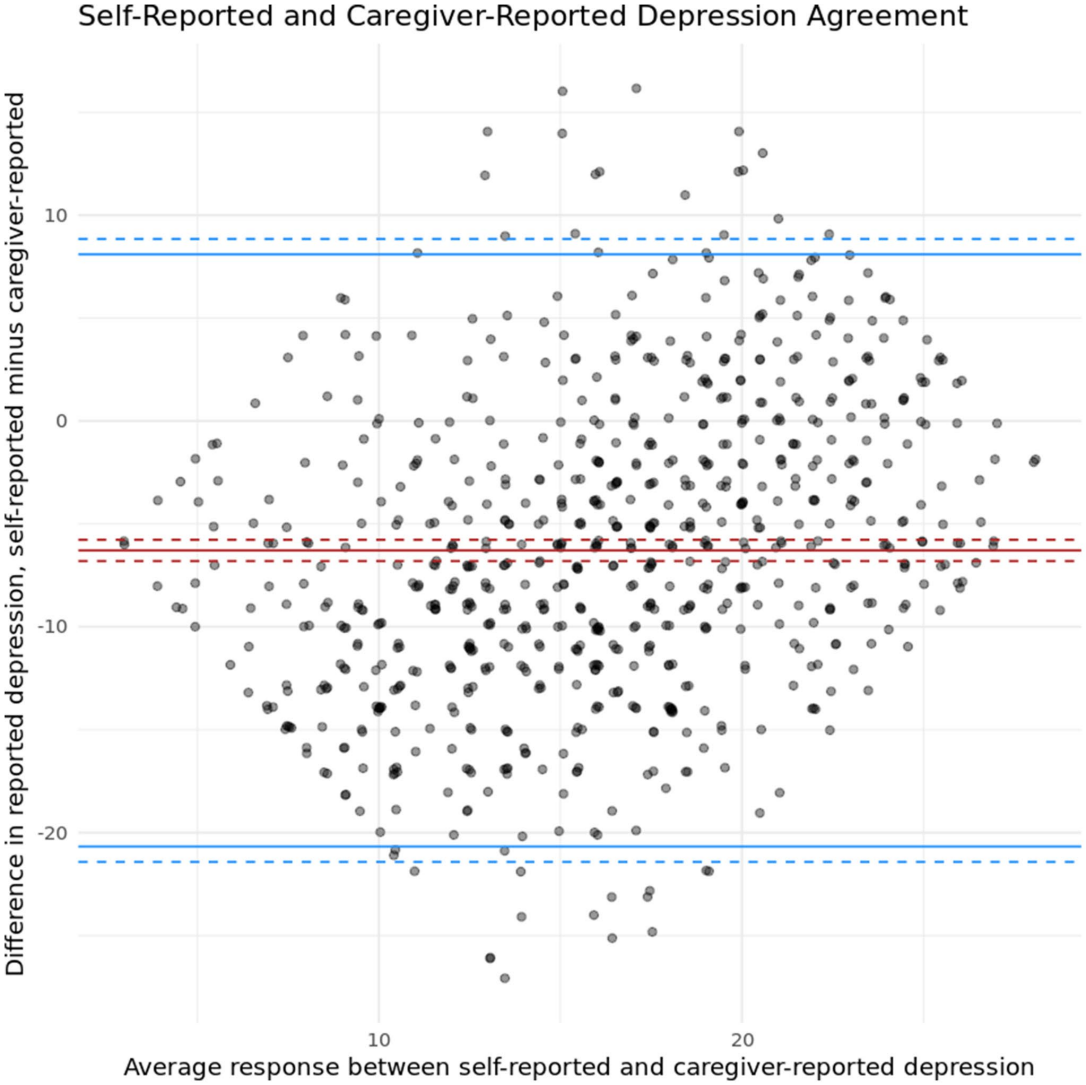
Bland–Altman plot showing agreement between self-reported and caregiver-reported depression symptoms.

**Figure 2:**
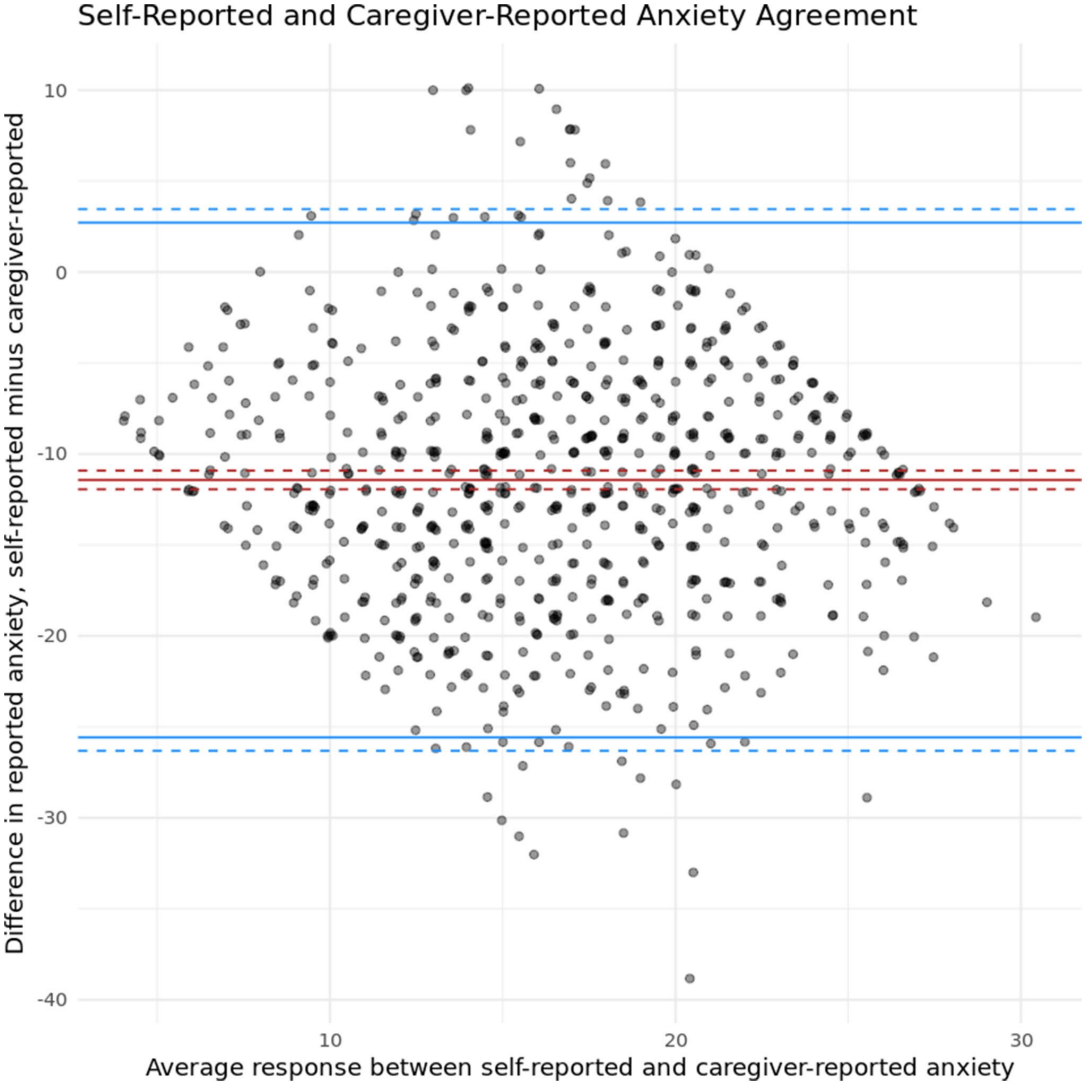
Bland–Altman plot showing agreement between self-reported and caregiver-reported anxiety symptoms.

To determine general trends in symptom severity agreement between caregivers and their loved one at intake, Lin’s concordance correlation coefficient was calculated between client self-reported depression symptoms and caregiver-reported depression symptoms. A statistically significant, moderate positive correlation was found between the two measures (*r* (770) = 0.215, *p* < 0.05, 95% CI [0.1714, 0.2585]). Similar, though slightly lower, results were found between client self-reported anxiety symptoms and caregiver-reported anxiety symptoms (*r* (761) = 0.124, *p* < 0.05, 95% CI [0.1714, 0.2585]). Note that Lin’s concordance correlation coefficient contains a penalty that scales with the difference in mean responses. As these ratings are on different scales, this provides an extremely conservative lower bound on agreement. These results indicate that while clients and caregivers have a moderate agreement level regarding symptom severity for both anxiety and depression, they are not a one-to-one match and some discrepancies are present. Figures 1, 2 present Bland–Altman plots for depression and anxiety, respectively, to probe these discrepancies for heterogeneity across symptom severity. In both cases, we observe more variation in agreement near mean levels of symptom severity, and more agreement at more extreme (high or low) levels of symptom severity.

**Figure 3:**
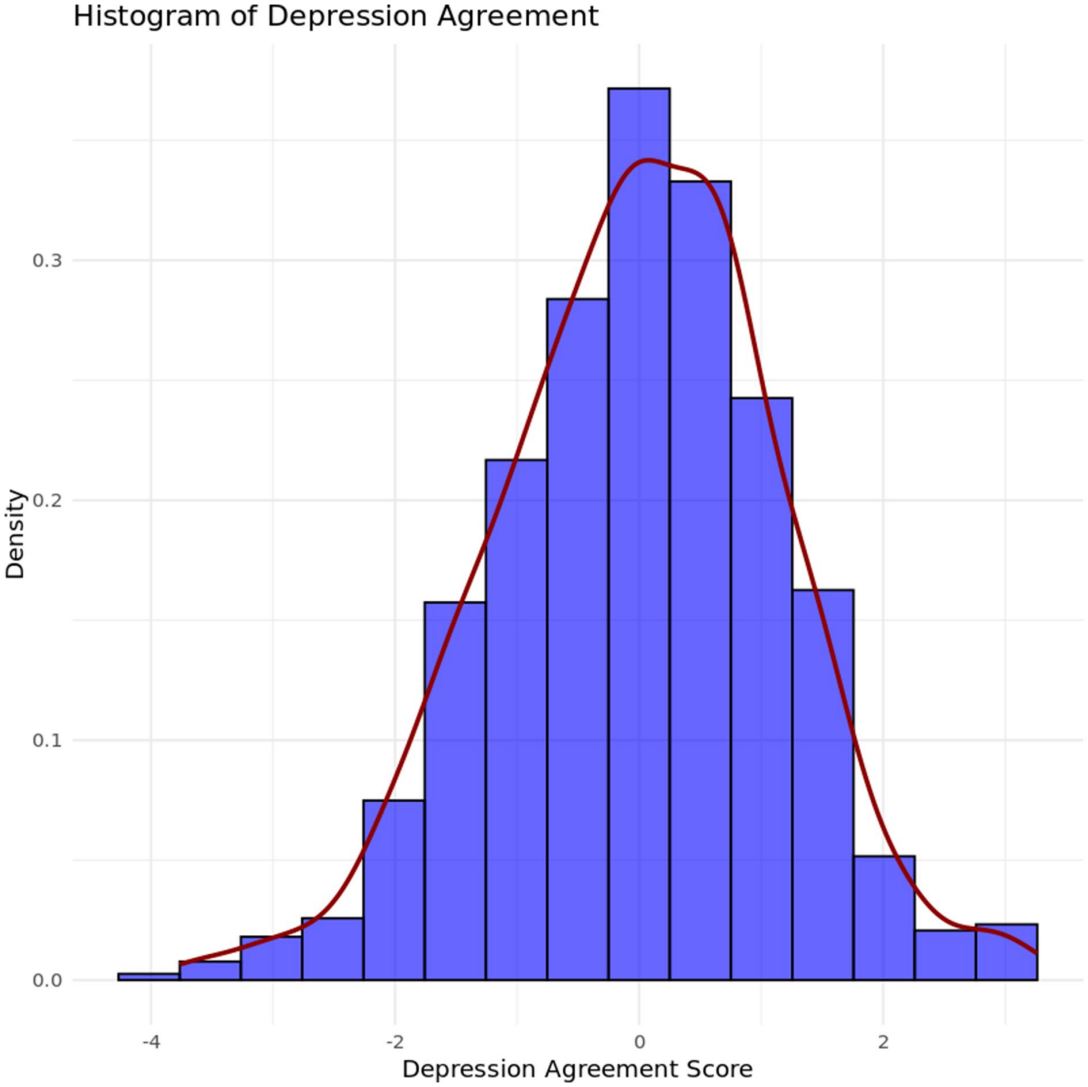
Histogram of agreement scores on depression symptoms between caregivers and clients.

**Figure 4:**
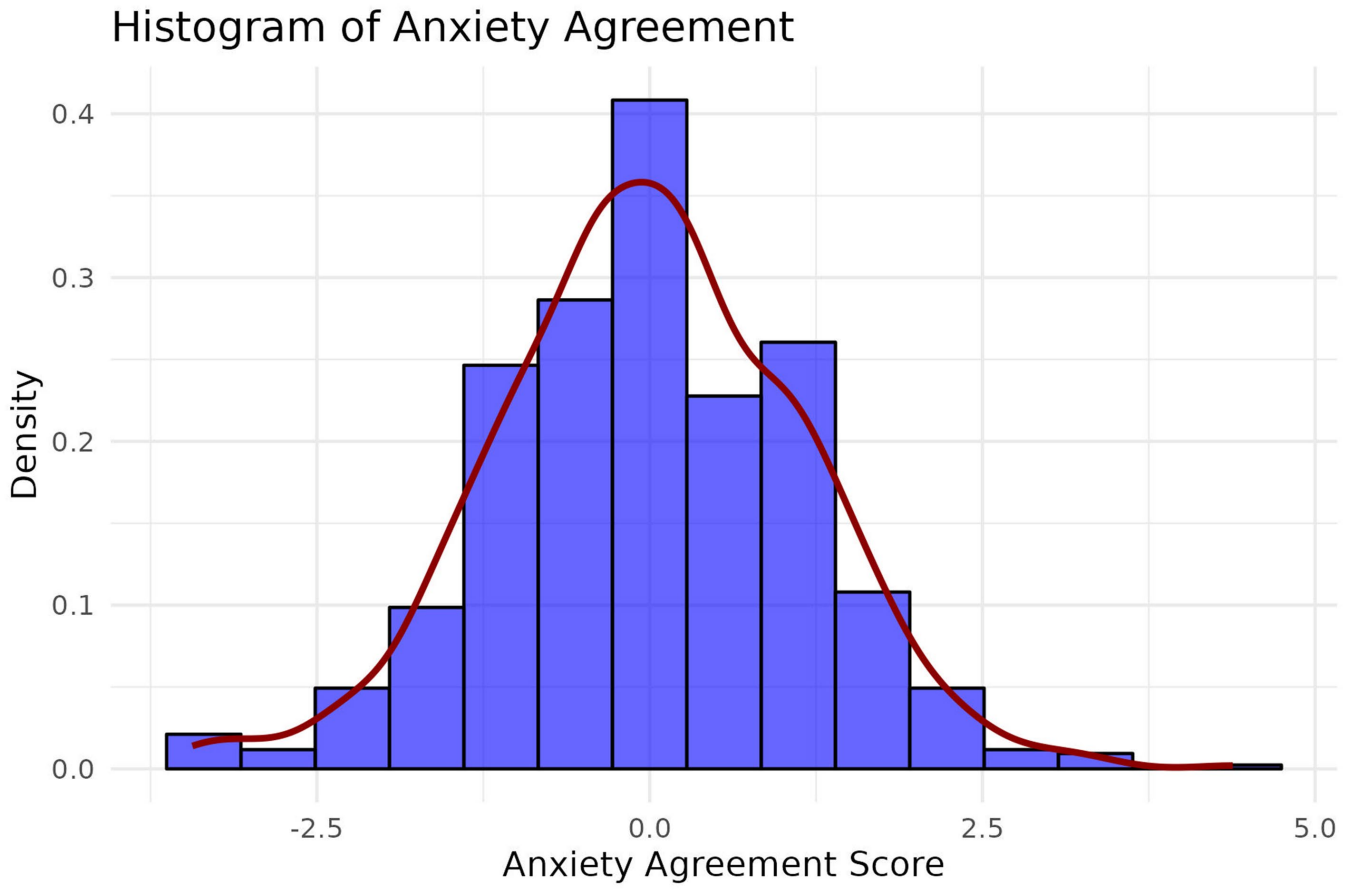
Histogram of agreement scores on anxiety symptoms between caregivers and clients.

Presenting agreement in a slightly different way, we first find *z*-scores of both self-reported and caregiver-reported depression and anxiety scores and compute the difference. Figures 3, 4 show a histogram of these differences for depression and anxiety, respectively. At intake, the average difference in scaled depression scores was −0.02 indicating almost perfect agreement between clients and caregivers on average with a slight tip towards clients rating depression symptoms as more severe. Similarly, the average difference in scaled anxiety symptoms at intake was −0.001, indicating clients and caregivers were using corresponding locations on their respective scales.

#### 3.2.2 Agreement by demographic variables

Due to the number of follow-up regressions and subgroup analyses conducted, these analyses should be considered exploratory and interpreted with caution rather than as confirmatory tests.

A linear regression was run to determine the relationship between level of symptom agreement and client age. Age was a significant predictor of depression symptom severity agreement *b* = 0.039, *t* (770) = 2.79, *p* = 0.005, indicating that every one-year increase in age is associated with an increase in depression agreement scores of 0.039 points. In other words, the older a client was at the time of data collection, the more the score shifted to caregivers scoring the client’s depression symptoms as worse. While the overall model was significant, it only predicted 1% of the variance in agreement (multiple *R*^2^ = 0.010; adjusted *R*^2^ = 0.009). Similarly, age was a significant predictor of anxiety symptom severity agreement *b* = 0.036, *t* (761) = 2.603, *p* = 0.009, indicating that for every 1-year increase in age, anxiety agreement scores increased by 0.036 points. Again, this model only explained approximately 1% of the variance in agreement scores. While both cases were significant, effect sizes indicate that the significance may only be a statistical artifact of sample size, and not a clinically significant finding.

Three ANOVAs were run to assess the patterns of symptom agreement based on client race, gender, and neurodivergent identity. Due to the repeated testing, a Bonferroni correction was applied to each ANOVA test, with a new significance level of *p* = 0.0167.

The first ANOVA looked at patterns of depression agreement based on a client’s reported gender. A one-way ANOVA indicated a significant main effect of gender on depression symptom agreement levels *F* (2, 613) = 13.45, *p* < 0.001 and this finding remained significant after the Bonferroni adjustment. *Post-hoc* comparisons using a Tukey HSD test revealed significant differences between all three gender categories with the largest difference in mean agreement scores found between Male clients and Non-Binary/Other clients (*M* = 0.229 vs. *M* = −0.502). Similarly, a one-way ANOVA indicated a significant main effect of gender on anxiety symptom agreement levels *F* (2, 606) = 4.54, *p* = 0.011. This finding also remained significant after the Bonferroni correction. A *post-hoc* Tukey HSD test revealed that significant differences in anxiety agreement levels were found between male and non-binary clients, and female and non-binary clients, but not between male and female clients.

Next, we investigated the relationship between a client’s neurodivergent identity and levels of symptom agreement. Based on one-way ANOVAS, depression symptom agreement scores were not associated with a client’s neurodivergent identity (*F* (4, 580) = 0.67, *p* = 0.611). Anxiety symptom agreement was significantly associated with a client’s neurodivergent identity *F* (4, 573) = 2.92, *p* = 0.021, however this model only predicted approximately 2% of the variance, and therefore while statistically detectable, was not clinically meaningful.

Finally, we assessed the impact of client race on symptom agreement levels. Based on one-way ANOVAs, neither depression nor anxiety symptom agreement scores were associated with client racial categories (*F* (2, 587) = 0.35, *p* = 0.71; *F* (2, 582) = 0.58, *p* = 0.56).

#### 3.2.3 Family functioning and symptom agreement

To determine if the level of family functioning impacts symptom agreement, regressions were performed looking at overall symptom agreement for both anxiety and depression, as well as magnitude of disagreement (regardless of direction), and sign of disagreement (regardless of magnitude). A simple linear regression on family functioning and depression agreement indicated a statistically significant relationship between the two variables (*β* = −0.036, SE = 0.008, *t* (603) = −4.42, *p* < 0.001), where every additional point of family functioning predicts a 0.036 point reduction in agreement score. This linear model explains 3.1% of variance in agreement scores, meaning family functioning had a small but significant effect on depression agreement.

However, upon running a linear regression on the magnitude of disagreement and family functioning, and a logistic regression on the direction of disagreement (client-higher vs. caregiver-higher), family functioning scores were only significantly associated with the direction of disagreement *χ*^2^ (1) = 6.17, *p* = 0.013. The regression coefficient for family functioning was negative (*β* = −0.036, SE = 0.015, *Z* = −2.46, *p* = 0.014), indicating that as family functioning increases, the likelihood of caregivers rating their loved-one’s depression symptoms higher than the client themselves decreased.

Conversely, family functioning was not significantly associated with overall anxiety symptoms agreement (*F* (1, 591) = 0.43, *p* = 0.511), magnitude of symptom agreement (*F* (1, 591) = 0.49, *p* = 0.484), or direction of symptom agreement (*χ*^2^ (1) = 0.96, *p* = 0.328).

#### 3.2.4 Caregiver well-being at intake and symptom agreement

To explore whether caregiver well-being was associated with agreement between caregiver- and child-reported symptoms, two linear regressions were conducted using the WHO-5 Well-Being Index as a predictor. Higher caregiver well-being was very weakly associated with lower agreement on depression symptoms, (*b* = −0.004, *p* = 0.03, 95% CI [−0.008–0.000]), though the model only explained 1% of the variance (*R*^2^ = 0.01). No significant association was found between caregiver well-being and anxiety agreement, *b* = −0.002, *p* = 0.34. These findings suggest that caregiver well-being may play a limited role in shaping agreement on symptom severity, specifically for depression, however it is not a strong influence.

#### 3.2.5 Symptom agreement and treatment outcomes

Of the 763 caregiver–youth dyads included at intake, 221 (28.9%) also completed a discharge survey and were therefore included in the outcomes analyses. This analytic sample provides a meaningful window into treatment change, but the reduced proportion of available discharge data should be considered when interpreting results.

**Figure 5:**
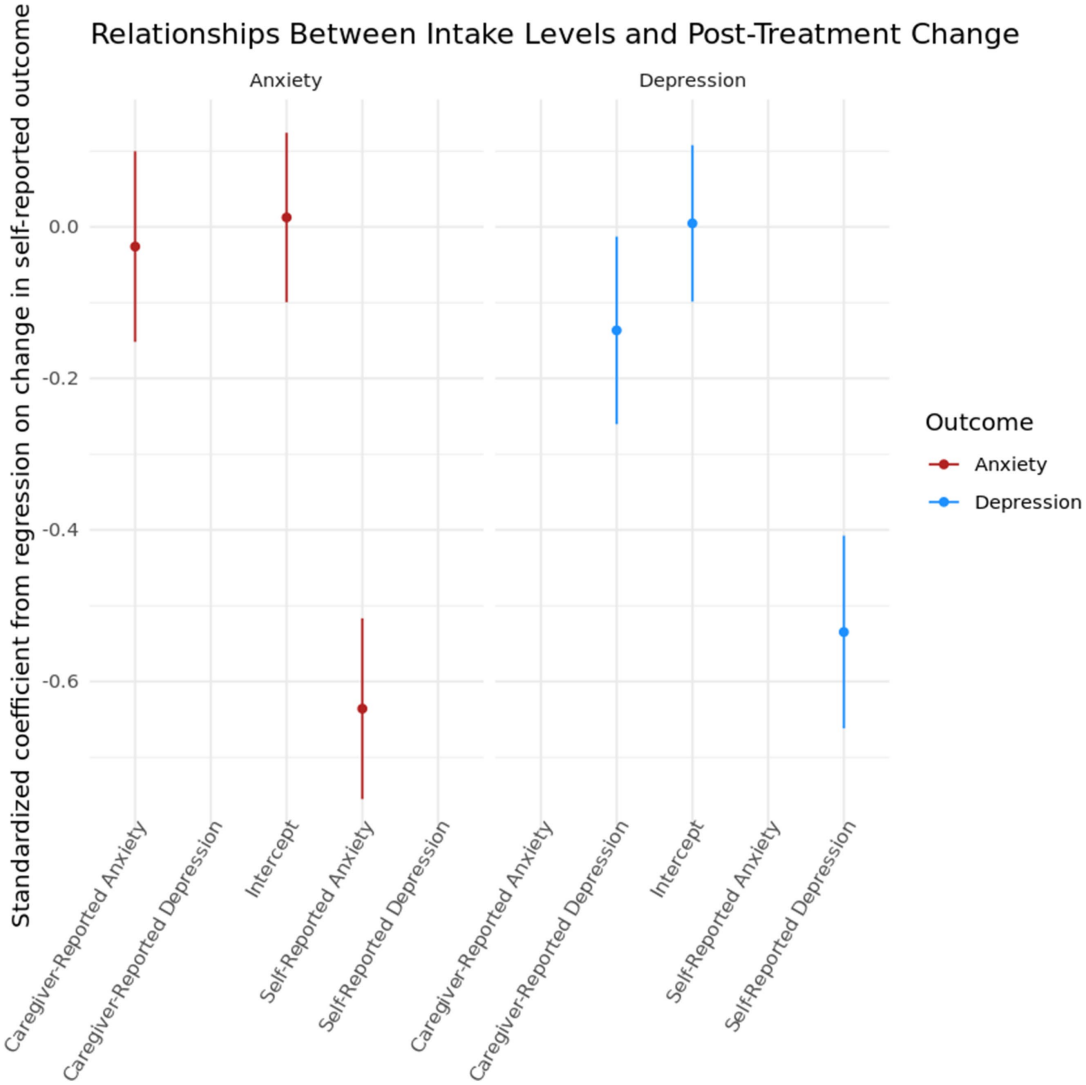
Coefficients from regressions of change in anxiety and depression on baseline levels of anxiety and depression. Anxiety and depression models were fit separately and are shown with 95% confidence intervals using heteroskedasticity-robust standard error corrections.

For both depression and anxiety, we fit a linear regression on the change in standardized differences in self-reported depression/anxiety on standardized measures of caregiver and self-reported depression/anxiety at intake. Figure 5 shows coefficients with 95% confidence intervals constructed from heteroskedasticity-robust standard errors from these regressions. In the left panel, we see that only the coefficient on self-reported anxiety is statistically distinguishable from zero (*β* = −0.636, 95% CI = [−0.755, −0.517]), indicating that individuals with higher levels of self-reported anxiety saw greater reductions in their self-reported anxiety levels post-treatment. For depression, both caregiver-reported depression levels (*β* = −0.137, 95% CI = [−0.260, −0.013]) and self-reported depression levels (*β* = −0.535, 95% CI = [−0.662, −0.408]) were statistically significant, though the predictive utility of the caregiver-reported level is smaller in magnitude than the self-reported level. We observe, similarly to anxiety, higher levels of self-reported depression are associated with higher reductions in self-reported depression post-treatment, and the magnitude of this effect is similar across both constructs. In both cases, despite there being some degree of disagreement between caregiver-reported and self-reported levels, the additional variance in the caregiver-reported levels explains little additional variance in the change in the self-reported outcome.

##### 3.2.5.1 Depression

**Figure 6:**
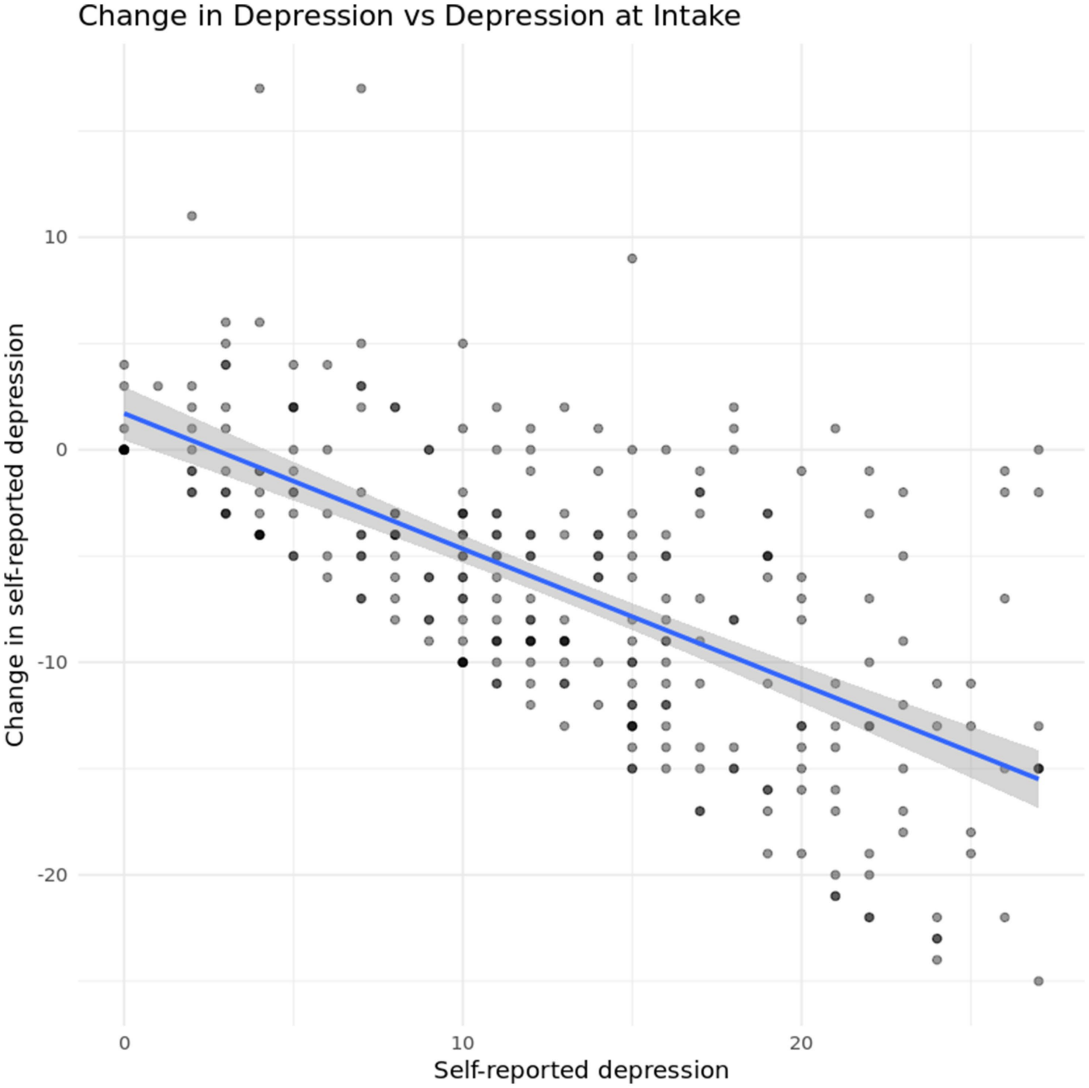
Change in depression vs. self-reported depression at intake.

One concern with the previous results is a potential regression-to-the mean effect. Figure 6 shows changes in self-reported depression plotted against self-reported depression at intake. In general, we see that self-reported depression decreases for most participants, regardless of their position on the scale, though the limits of the original scale impose limits on the observable variation in the outcome.

**Figure 7:**
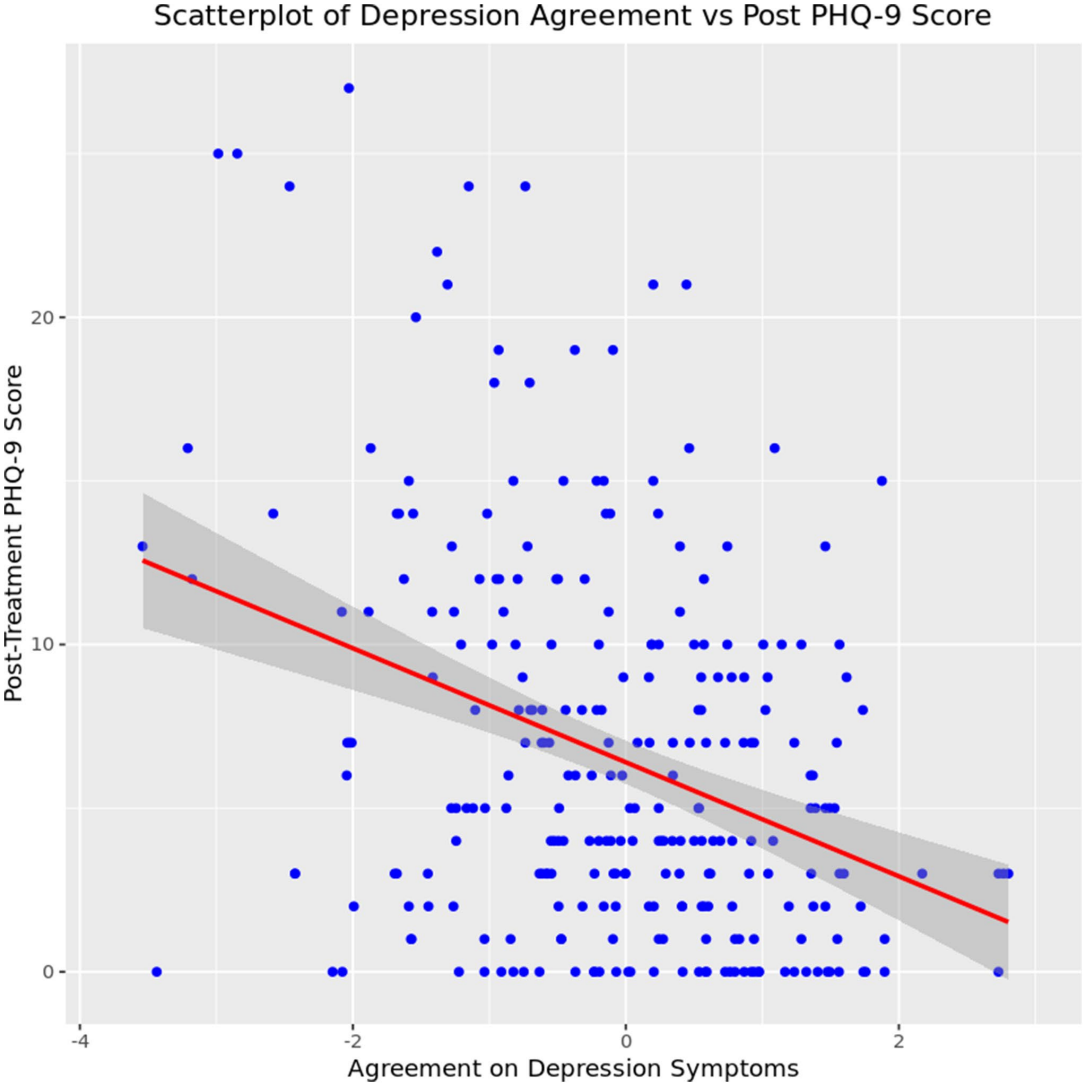
Relationship between caregiver-youth agreement on depression symptoms at intake and youth depression severity at discharge.

To investigate heterogeneity as a function of self-reported and caregiver-reported agreement, we look at a regression of average depression level at discharge on symptom agreement at intake. Results indicated a statistically significant relationship between depression symptom agreement at intake and depression symptoms at discharge (*F* (1, 219) = 27.15, *p* < 0.001,), and the model predicted 11.03% of the variance in depression symptoms at discharge. Additionally, the regression coefficient for depression agreement was significant (*B* = −1.64, SE = 0.32, *t* (219) = −5.21, *p* < 0.001) indicating that the higher a caregiver rates their child’s depression symptoms in relation to the client’s self-assessment, the less severe depression symptoms were at discharge, as seen in Figure 7.

While higher caregiver rated scores at intake were associated with lower client-reported depression symptoms at discharge, agreement level at intake was not significantly associated with likelihood of routine discharge. A logistic regression was run to determine whether overall depression agreement score, absolute depression agreement score (regardless of direction of disagreement), or the direction of agreement score (regardless of magnitude) were significant predictors of routine discharge, and found that none of the independent variables significantly predicted discharge type (*χ*^2^(3, *N* = 709) = 1.47, *p* = 0.689). Additionally, just caregiver ratings of client depression at intake did not have a significant association with likelihood of routine discharge (*t* (759.37) = 1.84, *p* = 0.066), indicating that once a client is in treatment, caregiver perception of symptoms does not seem to have an impact on treatment engagement.

Based on prior tests indicating that age, gender, and level of family functioning has an impact on depression symptom agreement, a final multiple linear regression was run to determine if age, gender, or family functioning have a mediating effect on depression symptoms at discharge. The overall model was statistically significant *F* (5,188) = 4.79, *p* = 0.00038, however it only accounted for ~11.3% of variance in depression scores. Depression symptom agreement at intake was the strongest predictor of depression scores at discharge, with caregivers scoring depression symptoms as more severe predicting lower post-treatment depression symptoms (*β* = −1.57, *p* < 0.001). Age, gender, and family functioning were not significant moderators in this model.

##### 3.2.5.2 Anxiety

**Figure 8:**
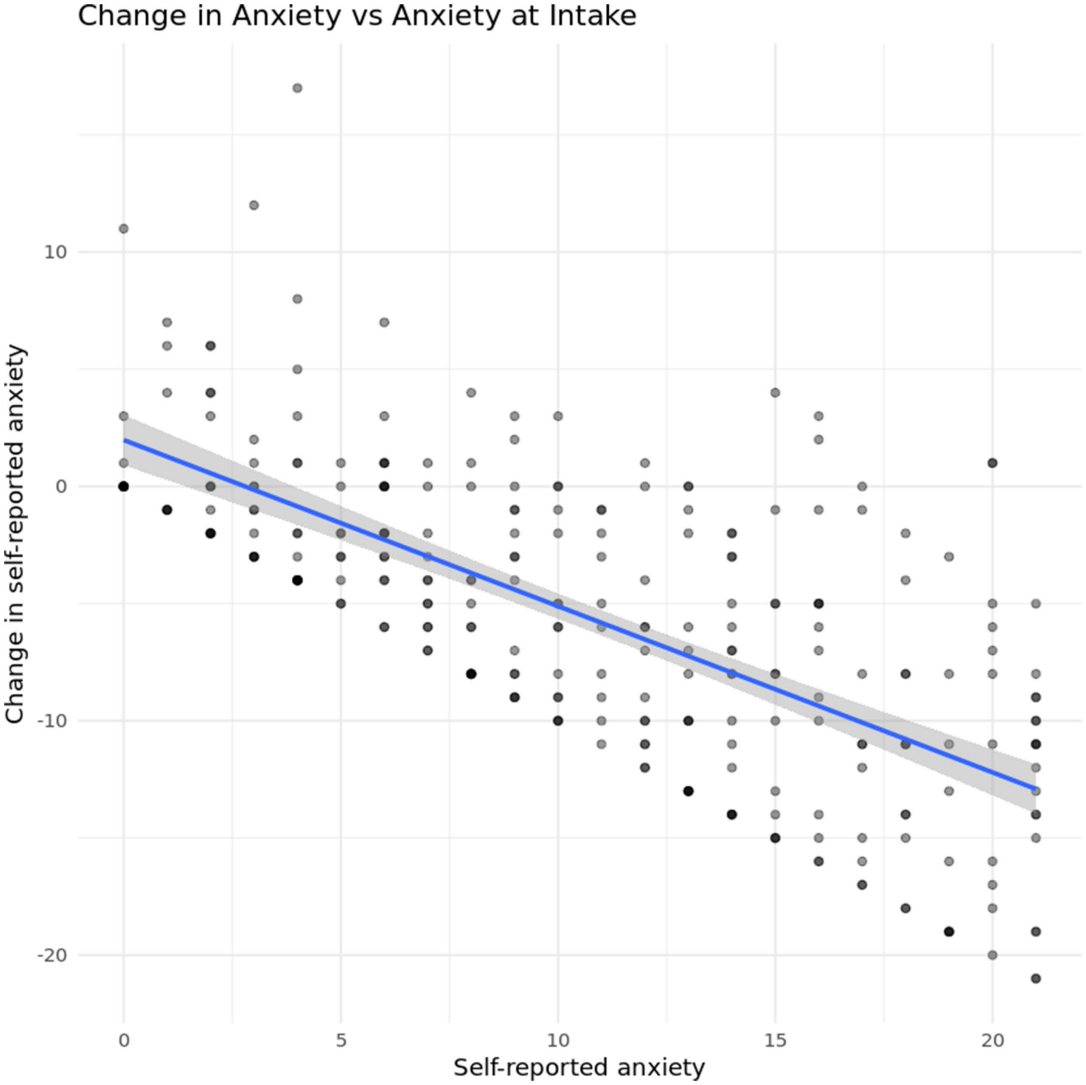
Change in depression vs. self-reported depression at intake.

As with depression, we first look for a regression-to-the mean effect. Figure 8 shows changes in self-reported anxiety plotted against self-reported anxiety at intake. In general, we again see that self-reported anxiety decreases for most participants, regardless of their starting position on the scale. The finite range of values achievable on the original scale still impose limits on the observable variation in the outcome.

**Figure 9:**
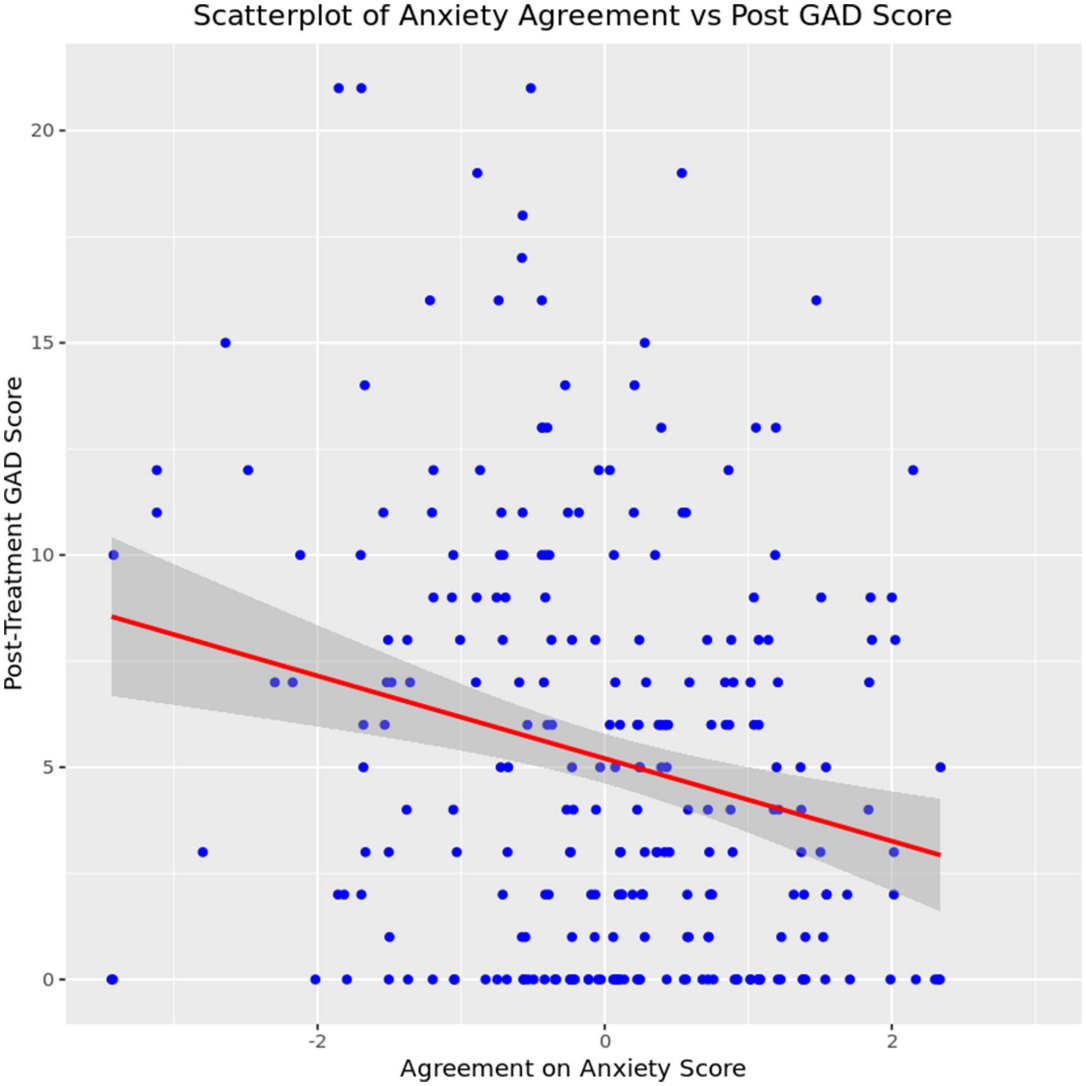
Relationship between caregiver-youth agreement on anxiety symptoms at intake and youth anxiety severity at discharge.

The first analysis for anxiety symptoms investigated the relationship between level of agreement on anxiety symptoms at intake and anxiety score at discharge. A linear regression showed a significant relationship between anxiety agreement at intake and symptom acuity at discharge *b* = −1.14, *t* (212) = −4.06, *p* < 0.001, indicating that for every 1-point increase in anxiety agreement score at intake, GAD-7 scores at discharge fell by −1.14 points, as seen in Figure 9.

Similar to the depression agreement scores, a logistic regression was run to evaluate the effect of anxiety agreement scores on likelihood of completing treatment. Independent variables were overall anxiety agreement, absolute magnitude of anxiety agreement (regardless of the direction), and the direction of anxiety agreement–whether caregivers ranked symptoms higher, or the client did (regardless of magnitude). Results of the logistic regression indicated that none of the independent variables had a significant association with treatment completion (*χ*^2^ (3, *N* = 647) = 1.61, *p* = 0.658). Additionally, caregiver ratings of anxiety symptoms at intake on their own did not have a significant association with routine discharge rates, indicating that the conclusion drawn for the depression scores remains consistent: once a client is in treatment, their agreement levels do not seem to have an impact on treatment completion.

Based on prior tests indicating that age and gender were both significantly associated with level of anxiety symptom agreement between clients and their caregivers, a multiple linear regression was run to control for both age and gender. The overall model was statistically significant (*F* (4,199) = 4.71,*p* = 0.001), and results indicate that when controlling for these demographics, anxiety symptom agreement at intake was the only significant predictor of anxiety symptoms at discharge (*β* = −1.08, *p* < 0.001). However, this model only accounted for ~8.7% of the variance in scores at discharge (*R*^2^ = 0.087, adjusted *R*^2^ = 0.068), making it a weak predictor of anxiety symptoms at discharge.

## 4 Discussion

The aim of this study was to examine client self-reported symptoms of depression and anxiety within a virtual intensive outpatient program in comparison to caregiver-reported assessments, exploring potential moderating variables and evaluating the relationship between agreement and treatment outcomes. Overall, we found modest agreement levels between clients and their caregivers when evaluating depression and anxiety symptoms. This pattern is consistent with prior research indicating only modest caregiver–child concordance on internalizing symptoms, likely reflecting differences in symptom observability and self-insight (De Los Reyes and Ohannessian, 2016; Korelitz and Garber, 2016). However, these average agreement levels concealed meaningful subgroup differences that have implications for clinical practice and future research.

In addition to the primary analyses, we conducted several exploratory regressions to gauge which demographic or family variables might warrant inclusion as covariates in outcome models. Because of the number of comparisons and lack of a preregistered confirmatory plan, these results were not corrected for multiplicity and should be interpreted as hypothesis-generating rather than confirmatory. Notably, gender emerged as a key moderator of agreement. Non-binary youth showed the largest level of discrepancy with their caregivers. Specifically, non-binary participants were more likely to rate their symptoms as more severe than their caregivers did. This finding echoes existing literature that highlights the unique mental health burdens faced by gender-diverse youth (Eisenberg et al., 2017). Additionally, age was significantly associated with level of disagreement with older clients reporting fewer symptoms relative to their caregivers’ ratings, for both anxiety and depression. However, age explained only 1% of the variance and is therefore unlikely to be meaningful. These exploratory findings should be interpreted as small effects that may reflect only statistical power rather than substantive influence.

While race and neurodivergent identity were investigated as potential moderators of agreement levels, neither category showed any significant differences. While no significant associates were found for race or neurodivergent identity, these null results should be interpreted with caution. The sample was relatively homogeneous with respect to race/ethnicity and missing data was substantial. Similarly the neurodivergent identity categories were broad, which may have obscured more specific patterns.

Results also indicated that caregiver ratings of family functioning were significantly associated with disagreement levels on depression symptoms. Specifically, in families reporting higher functioning, caregivers were less likely to overestimate client symptom severity compared to the client’s own report. This may suggest that higher levels of family functioning are associated with greater agreement between caregiver and client perceptions of symptom severity. This aligns with family systems theory, which emphasizes that higher-functioning families often display stronger communication and shared understanding of stressors (Erdem and Safi, 2018). Agreement in symptom perception may therefore reflect healthier relational dynamics rather than only observational accuracy. However, this relationship did not hold for anxiety symptoms, suggesting that internalizing symptoms may vary in how observable or interpretable they are within family systems.

Contrary to prior literature that found that caregiver distress can bias caregiveral proxy ratings of child mental health (Ehrlich et al., 2011), no significant relationship between caregiver well-being and level of disagreement was found within this sample. This may be due to the fact that there was not much deviation in well-being scores between caregivers, and therefore the spread was not comprehensive enough to identify a relationship. Alternatively, caregiver well-being, while important to consider in other aspects of treatment, may not sway the perception of their loved-ones symptoms. This is an area that warrants further exploration. Given the limited variance in caregiver well-being scores, it is possible that this study lacked the sensitivity to detect meaningful effects.

Finally, we examined the relationship between caregiver-client agreement at intake and treatment outcomes. In general, clients with higher self-reported depression and anxiety had better outcomes (operationalized as larger reductions in self-reported depression and anxiety), and after controlling for self-reported depression and anxiety at intake, caregiver-reported levels of depression and anxiety explained little additional variance in the outcome. Prior research has similarly found that client self-reports often show stronger associations with treatment response than parent ratings, particularly for internalizing symptoms (Hawley and Weisz, 2003; Ferdinand et al., 2006).

Interestingly, dyads in which caregivers scored client symptoms as more severe were associated with lower severity symptoms at discharge as measured by the PHQ-9 and GAD-7 for both anxiety and depression. At first glance, this pattern may appear counterintuitive, yet it aligns with several possible mechanisms. One plausible explanation is caregiver engagement and advocacy: When caregivers perceive distress as high, they may be more motivated to advocate for services, encourage adherence, and partner with providers, thereby indirectly facilitating client improvement. Previous work suggests that greater caregiver concern or involvement can enhance treatment adherence and engagement, leading to better clinical improvement (Hawley and Weisz, 2005). From a family systems perspective, this could reflect a dynamic in which caregivers’ acknowledgement of distress validates the client’s experience, increasing motivation to engage in treatment (Erdem and Safi, 2018). Additionally, caregiver perception of high distress may allow the client to feel safer expressing and exploring emotion due to the expectation of emotion already being present (De Los Reyes et al., 2013; Hawley and Weisz, 2003). This exploration of distress may then result in better understanding of symptoms and willingness to change.

Another interpretation is that heightened caregiver concern may shape treatment processes themselves—for example, prompting earlier recognition of problems, increasing caregiver participation in family sessions, or leading to more consistent use of the caregiver portal. Although such variables were not directly measured in the current study, future work should examine whether caregiver-rated severity functions as a proxy indicator for family engagement.

Despite the improved outcomes when caregivers scored symptom severity as higher, agreement level itself was not significantly associated with the likelihood of routine discharge for either depression or anxiety. This null result is noteworthy as it suggests that while caregiver perceptions may influence the severity of symptoms and the trajectory of symptom change, they do not appear to influence whether clients complete treatment in a virtual intensive outpatient program (IOP) setting. One possible explanation is that the structural supports inherent to IOP reduce the impact of informant agreement in predicting treatment continuation. Alternatively, agreement may be more critical at intake, influencing early engagement and symptom recognition, but less influential once clients are admitted into care.

These findings underscore the importance of assessing both client and caregiver symptom reports at intake. Specifically, caregiver perceptions of higher severity were associated with greater symptom improvement, suggesting that engaging caregivers early and validating their concerns may promote treatment buy-in and adherence. Prior work has emphasized that explicitly addressing informant discrepancies in session can improve therapeutic alliance and goal consensus (De Los Reyes, 2011; Hawley and Weisz, 2005). However, discrepancies, particularly among non-binary youth, highlight the need for clinicians to facilitate structured conversations about symptom experience to foster mutual understanding and goal alignment. The counterintuitive nature of this finding highlights the complex role of caregiver perception. It may be that caregivers who recognize and emphasize their client’s distress are more likely to advocate for services, encourage adherence, and partner with providers, thereby indirectly facilitating improvement.

## 5 Limitations and future research

Due to the amount of missing and skewed demographic data (which leaned heavily toward white, neurotypical, female participants) this dataset has limited generalizability, and conclusions should not be applied to all individuals with mental health concerns. Furthermore, several demographic and outcome variables were collapsed into broader categories to reduce small cell sizes and ensure statistical stability. For example, gender identities beyond male and female were combined into an “Other/Non-binary” category, and racial identities outside of White and Black were combined into “Other.” Similarly, discharge reasons were collapsed into “routine” versus “non-routine” to address small subgroups such as administrative discharge or referral to higher levels of care. While this approach allowed analyses to proceed without risk of overfitting or disclosure, it also limited the granularity with which subgroup differences and discharge patterns could be interpreted. Future work with larger and more diverse samples should retain finer categories and apply analytic methods designed to handle sparse cells (e.g., penalized regression or Bayesian models) to better capture heterogeneity across groups. Future research should focus on gathering a representative sample across demographics to assess how these results may differ depending on individual identity.

This study may also have been swayed by selection bias, where families willing to participate in caregiver surveys may differ systematically compared to those who did not fill out the survey. Only a small proportion of families completed the caregiver survey, and among those, fewer than half had discharge data available, raising the possibility of non-random attrition influencing outcome estimates. This was partially demonstrated by the skewed distribution in demographics that did not align with overall population demographics within the treatment center. Future research should find ways to require the caregiver survey, or otherwise randomly select individuals to complete the caregiver survey to reduce the risk of selection bias.

Caregiver and youth surveys were collected on slightly different schedules and with different recall windows (7-day vs. 14-day). These decisions allowed analyses to proceed without risk of overfitting or disclosure but limited the granularity with which subgroup differences, discharge patterns, and agreement estimates could be interpreted. Future work should aim to retain finer categories, synchronize data collection more tightly, and use analytic methods designed to handle sparse cells (e.g., penalized regression or Bayesian models) to better capture heterogeneity across groups. It is also important to note that agreement estimates may be influenced by the use of different instruments and informant types. Clients and caregivers completed distinct measures, and while *z*-standardization facilitated comparability, differences in response format, interpretation, and construct coverage may constrain the accuracy of cross-informant agreement.

Additionally, this study was limited to individuals who were already admitted to high acuity mental healthcare, which restricts the generalizability of this outcome. The question that needs to be answered may not be how agreement and disagreement impact treatment outcomes, but how they impact access to care in the first place. Further research should examine client and caregiver symptom agreement in the general population and track individuals longitudinally to determine who ends up in treatment and who does not.

Finally, as several authors are employed by the treatment program studied, there is a potential for bias in the interpretation. However, the outcomes reported were based on client self-report rather than clinician or staff ratings, reducing the risk that author employment influenced results.

## Data Availability

For the sake of patient confidentiality, the dataset analyzed for this study is not publicly available. Please contact the authors to request access to the dataset.
